# Serum PFAS in Aircraft Rescue and Firefighting (ARFF) Firefighters From Six U.S. Airport Fire Departments

**DOI:** 10.1002/ajim.70084

**Published:** 2026-04-26

**Authors:** Miriam M. Calkins, Yiwen Liu, Gavin Horn, Antonia M. Calafat, Judith M. Graber, Shawn C. Beitel, Alberto J. Caban‐Martinez, Julianne Cook Botelho, Randy Krause, Rob Mathis, Jeff Hughes, Casey Grant, Jaclyn M. Goodrich, Amy Nematollahi, Alesia M. Jung, Alissa D. Coleman, Sally Littau, Brooke A. Hawkes, Alexander C. Mayer, Jefferey L. Burgess

**Affiliations:** ^1^ National Institute for Occupational Safety and Health, Centers for Disease Control and Prevention Cincinnati Ohio USA; ^2^ Department of Epidemiology and Biostatistics University of Arizona Mel and Enid Zuckerman College of Public Health Tucson Arizona USA; ^3^ Fire Safety Research Institute, UL Research Institutes Columbia Maryland USA; ^4^ National Center for Environmental Health, Centers for Disease Control and Prevention Atlanta Georgia USA; ^5^ Department of Biostatistics and Epidemiology Rutgers the State University of New Jersey, Piscataway New Jersey USA; ^6^ Department of Community, Environment and Policy University of Arizona Mel and Enid Zuckerman College of Public Health Tucson Arizona USA; ^7^ Department of Public Health Sciences University of Miami Miller School of Medicine Miami Florida USA; ^8^ Port of Seattle Fire Department Seattle Washington USA; ^9^ Port of Portland Fire Department Portland Oregon USA; ^10^ Orange County Professional Firefighters, IAFF Local Tustin California USA; ^11^ DSRAE LLC Belmont Massachusetts USA; ^12^ Department of Environmental Health Sciences University of Michigan School of Public Health, 1415 Washington Heights Ann Arbor Michigan USA; ^13^ Exponent Inc. Menlo Park California USA

**Keywords:** aircraft rescue and firefighting, airport, biomonitoring, environmental exposures, firefighters, occupational exposures, Per‐ and polyfluoroalkyl substances, PFAS

## Abstract

**Introduction:**

Use of aqueous film‐forming foam (AFFF) is a source of exposure to per‐ and polyfluoroalkyl substances (PFAS) for firefighters working in aircraft rescue and firefighting (ARFF) settings. However, data characterizing the association between serum PFAS concentrations and exposure risk factors for ARFF firefighters are limited.

**Methods:**

In this cross‐sectional study, ARFF firefighters (*N* = 193) from six U.S. commercial airports provided serum for quantification of nine PFAS and completed a survey in 2019‐2020. A drinking water sample from each fire station was also analyzed for 29 PFAS. Serum PFAS concentrations were compared with demographically‐similar participants from the National Health and Nutrition Examination Survey (NHANES) 2017‐March 2020. Multivariable linear regression was used to identify factors associated with serum PFAS concentrations.

**Results:**

Geometric mean serum concentrations of perfluorohexane sulfonic acid (PFHxS), perfluorooctane sulfonic acid (PFOS) branched isomers, and perfluoroundecanoic acid (PFUnDA) were statistically higher in ARFF firefighters compared with NHANES participants. PFAS were detected in tap water at three fire departments, but only one department was characterized by detection of select PFAS (perfluorooctanoic acid (PFOA), PFOS, and PFHxS) in both water and serum. Past employment, detection of PFAS in drinking water, and age were positively associated with select PFAS concentrations; a recent change in workplace AFFF behavior or practice, female sex, and Black race exhibited inverse associations.

**Conclusions:**

Participants reporting changes in workplace behavior, policy, or practice had lower summed PFAS concentrations, suggesting these measures may help reduce exposure. Continued research is needed to evaluate exposure reduction strategies for firefighters, particularly those working in ARFF settings.

## 1 Introduction

Per‐ and polyfluoroalkyl substances (PFAS) are a prominent synthetic chemical group of concern for firefighters [1, 2, 3]. PFAS are widely used across industries and in consumer products due to their versatile applications [4], although their essentiality of use and availability of replacement technologies vary [5, 6]. For firefighters, known and potential PFAS (primarily perfluoroalkyl carboxylic acids [PFCAs] and perfluorosulfonic acids [PFSAs]) exposure pathways include aqueous film forming foam (AFFF) [7], personal protective equipment (PPE) [8, 9, 10, 11, 12], fire station dust [13], products of incomplete combustion [14], and contaminated drinking water [15, 16]. We previously observed higher concentrations of certain long‐alkyl‐chain PFAS in the blood of municipal United States (U.S.) firefighters compared with data from demographically‐similar members of the general U.S. population in the National Health and Nutrition Examination Survey (NHANES) [17, 18], and in firefighters compared with other essential workers in Arizona [19]. Studies of other geographic locations [20, 21], different firefighting subgroups [22, 23, 24], and firefighters performing specific AFFF training tasks [20, 25] have also demonstrated elevated PFAS in some firefighting populations.

In the U.S., AFFF has been used extensively at aircraft rescue and firefighting (ARFF) facilities due to federal requirements for commercial airports to purchase AFFF that is military specification (MILSPEC) compliant (14 CFR 139.317), but AFFF may also be used by municipal fire departments, fire response training institutions, and Department of Defense (DoD) installations. AFFF consists of PFAS, hydrocarbon surfactants, solvents, inorganic salts, corrosion inhibitors, and water. When appropriately applied by trained firefighters, these mixtures have the ability to spread in a blanket‐style formation, making it very effective against Class B flammable liquid fires [26]. PFAS were first used in AFFF in the 1960s with formulations predominantly including long‐alkyl‐chain perfluoroalkyl sulfonates, such as perfluorooctane sulfonic acid (PFOS) and perfluorohexane sulfonic acid (PFHxS) [25, 27, 28]. The formulation of AFFF has changed over time to include more short‐alkyl‐chain fluorotelomer‐based PFAS, but many of the original AFFF products have a long shelf‐life of up to 25 years. As a result, older products containing long‐alkyl‐chain PFAS (e.g. PFOS and PFHxS), purchased prior to recent changes in formulation, may exist in stockpiles available for continued use [28] at fire departments and airports across the U.S., despite changes in manufactured AFFF formulation. Even with the long shelf‐life, legislative and voluntary efforts to replace AFFF with fluorine‐free foam (“F3”) alternatives in the U.S. are well underway through State‐led regulations restricting use, release, and storage of AFFF; changes to the MILSPEC list in 2023 that allowed “F3”; and fire department‐led initiatives [25].

In a large survey of firefighters in the U.S. DoD, PFAS were consistently detected in serum [29]. In training environments, serum PFAS concentrations increased in Finnish firefighters after AFFF use [25], while in municipal departments in Australia, firefighters hired after use of AFFF was discontinued had lower PFAS serum concentrations than their colleagues when controlling for age and job tenure [20]. AFFF has also contaminated drinking water sources at multiple locations in the U.S. [30] with the potential for contamination of drinking water at fire stations, as well as the drinking water of the communities where these firefighters may reside.

In addition to exposure to AFFF, firefighters may potentially be at increased risk of exposure to PFAS through their turnout gear, station dust, and products of incomplete combustion. PFAS are integrated into textiles and used in liquid and stain resistant coatings for clothing [31], including firefighter turnout gear ensemble designed to be worn during firefighting operations [9, 32]. First integrated into turnout gear in the 1970s [25] as part of the thermal liner and as a durable water repellant (DWR) coating, the specific PFAS used in these products have changed over time. Data from studies of currently or recently manufactured products nearly universally identified PFAS across textiles used in turnout gear, with higher concentrations in the moisture barrier and outer shell than in the thermal liner [9]. In an assessment by the National Institute of Standards and Technology, PFAS quantities in the highest concentrations included the fluorotelomerization‐derived precursor PFAS such as 6:2 fluorotelomer methacrylate, 6:2 fluorotelomer alcohol, and 6:2 fluorotelomer sulfonic acid [9]. Concentrations of PFAS extractable from turnout gear materials increase when textiles were subjected to abrasion, weathering, and elevated temperature, and decrease following laundering [8]. Exposure to PFAS from turnout gear textiles may occur through direct contact with the gear as well as contamination of the work environment [11, 12]. Young et al. [13] documented the presence of PFAS in fire station dust and reported higher concentrations of total fluorine as well as some PFAS in turnout gear storage rooms. PFAS are also present in upholstery, insulation, electronics, cleaning products, surface coatings, and other common products [33]. Specific to fire scenes, exposure to heat can mobilize PFAS and PFAS precursors [34]. Studies characterizing the destruction efficiency of thermal treatment of PFAS during waste incineration indicate the presence of products of incomplete combustion at temperatures relevant to fire response settings such that PFAS may be present in the air during and following fire response activities [14]. However, the extent of exposure and toxicokinetic behavior, including the amount that enters the bodies of firefighters, from either their gear, dust at fire stations, combustion byproducts, or AFFF, is not known.

PFAS are known to adversely affect health. Specifically, reported toxicity of PFAS exposure in humans includes increased risk of some cancer types, pregnancy‐induced hypertension and preeclampsia, increases in cholesterol, decreases in birth weight, thyroid effects, ulcerative colitis, and reduced antibody response to some vaccines, as well as alterations in hepatic enzymes [35, 36, 37] Some of these diseases and conditions have also been reported in excess among firefighters, including urogenital cancers [38, 39, 40], increases in serum cholesterol [41], altered immune response and increased respiratory effects [42], and reproductive toxicity [43]. However, the extent to which PFAS exposures contribute to these health effects in firefighters is not known.

In 2023, the World Health Organization's International Agency for Research on Cancer (IARC) classified perfluorooctanoic acid (PFOA) as carcinogenic to humans (Group 1) based on sufficient evidence for cancer in animals and strong mechanistic evidence (e.g. via epigenetics and immunosuppression) in exposed humans, and PFOS as possibly carcinogenic to humans (Group 2B) based on strong mechanistic evidence from the same categories [44]. Epigenetic changes associated with PFAS have been documented in firefighter populations, including positive associations between accelerated epigenetic age and PFHxS, linear isomer of PFOA, and PFOS branched isomers [45]. In this same study, PFOA branched isomers, linear isomer of PFOS (n‐PFOS), perfluorononanoic acid (PFNA), perfluorodecanoic acid (PFDA), and perfluoroundecanoic acid (PFUnDA) were associated with differentially methylated loci and regions. Long‐alkyl chain PFAS (e.g., PFOA, PFOS, PFHxS) bind to proteins and bioaccumulate in lung, kidney, liver, and other organs, persisting for a long time in the body, with 3‐ to 8.5‐year serum half‐lives [46, 47, 48].

In 2016, the Fire Fighter Cancer Cohort Study (FFCCS) [49], a community‐engaged multicenter prospective cohort study designed to evaluate firefighter exposures and health effects with a primary focus on cancer, was established. For the current analysis, we evaluated serum concentrations of select PFAS in ARFF firefighters, collected survey information on risk factors for PFAS exposure, and collected drinking water samples from the participating fire stations. We hypothesized that ARFF firefighters will have higher concentrations of PFAS in serum quantified by the National Center for Environmental Health (NCEH) than seen in the general population, and that increased PFAS concentrations would be associated with increased exposure to AFFF, workplace procedures or practices during AFFF use, and contaminated fire station drinking water.

## 2 Methods

Incumbent firefighters working in six ARFF departments or stations (labeled as Departments A‐F) from six states in the U.S., were enrolled into the FFCCS in 2019 and 2020 after completing informed consent. At the time of enrollment, participants provided blood and urine samples for analysis of biomarkers of exposure and effect. The study participants also completed the FFCCS enrollment survey and an additional survey specific to AFFF use and other possible PFAS exposure sources. A point‐of‐use tap‐water sample was collected from the main drinking water source for each department. More details on the FFCCS enrollment process are available in a previous publication [50].

The enrollment survey was developed by the FFCCS Data Coordination Core for epidemiologic analyses and includes information on demographics, work history, health and behavior factors, and medical diagnoses. [50] Additional survey components are often developed for firefighter sub‐groups enrolled on the FFCCS to address specific considerations relating to work practices, exposure, and health. For this study, a survey consisting of potential PFAS‐related risk factors was developed to include information on potential use and contact with AFFF, time in PPE, and potential non‐firefighting PFAS exposures.

Blood samples were collected by qualified personnel upon enrollment following standard precautions. Blood was collected in red top tubes (Beckton, Dickinson and Company) for quantification of PFAS as previously described [18], with additional blood collected for microRNA, DNA methylation, and potential future analysis. Blood was shipped on ice (but not frozen) overnight to the University of Arizona and processed upon arrival. The serum tube was centrifuged at 1000–1300×*g* for 15 min, and the serum supernatant was stored in 0.5–1.0 mL aliquots and frozen at −80°C for long‐term storage until use. Frozen serum was shipped overnight on dry ice to the NCEH laboratory of the U.S. Centers for Disease Control and Prevention (CDC) for analysis. Serum was analyzed using isotope dilution tandem mass spectrometry as previously described [45, 51] for nine PFAS: PFHxS, n‐PFOS, sum of branched isomers of PFOS (perfluoromethylheptane sulfonic acid isomers (sm‐PFOS)), linear PFOA (n‐PFOA), branched isomers of PFOA (Sb‐PFOA), PFNA, PFDA, PFUnDA, and 2‐(N‐methyl‐perfluorooctane sulfonamido) acetic acid (MeFOSAA). The limit of detection (LOD), calculated from five repeated measurements of low‐level standards spiked onto serum as 3S_0_, where S_0_ is the standard deviation as the concentration approaches zero, for all PFAS was 0.1 ng/mL [51]. The analysis of deidentified specimens at the CDC laboratory was determined not to constitute engagement in human subjects research.

Water samples from each ARFF department were collected from a kitchen faucet or similar primary source of drinking water in a screw‐cap 250 mL polypropylene bottle, preserved with Trizma at 5.0 g/L. Each sample was accompanied by a field blank. Department C had a filtered water tap used for drinking water which was evaluated along with a non‐filtered sample from the kitchen sink. The water samples and field blanks were sent to American Water's research laboratory at 2°C–8°C and analyzed for PFAS within 14 days of shipping using an in‐house direct injection liquid chromatography‐tandem mass spectrometry method. Internal standards and surrogates were used for the PFAS being tested: perfluorobutanoic acid (PFBA), perfluoropentanoic acid (PFPeA), perfluorohexanoic acid (PFHxA), perfluoroheptanoic acid (PFHpA), PFOA, PFNA, PFDA, PFUnDA, perfluorododecanoic acid (PFDoA), perfluorotridecanoic acid (PFTrDA), perfluoroetradecanoic acid (PFTeDA), perfluoropropane sulfonic acid (PFPrS), perfluorobutane sulfonic acid (PFBS), perfluoropentane sulfonic acid (PFPeS), PFHxS, perfluoroheptane sulfonic acid (PFHpS), PFOS, perfluorononane sulfonic acid (PFNS), perfluorodecane sulfonic acid (PFDS), 4:2 fluorotelomer sulfonic acid (4:2FTS), 6:2 fluorotelomer sulfonic acid (6:2FTS), 8:2 fluorotelomer sulfonic acid (8:2FTS), 10:2 fluorotelomer sulfonic acid (10:2FTS), hexafluoropropylene oxide dimer acid (GenX), 4,8‐dioxa‐3H‐perfluorononanoic acid (ADONA), perfluoro‐4‐methoxybutanoic acid (PFMBA), perfluorooctane sulfonamide (FOSA), potassium 9‐chlorohexadecafluoro‐3‐oxanonane‐1‐sulfonate (9Cl‐PF3ONS), and 11‐chloroperfluoro‐3‐oxaundecanesulfonic acid (11Cl‐PF3OUdS). PFAS water concentrations are presented in ppt (pg/mL). Minimum detection limits (MDLs) ranged from 1 to 5 ppt, depending on the PFAS.

### 2.1 Statistical Analysis

Population demographics and occupational characteristics for ARFF firefighters were derived from FFCCS survey response data. Categorical variables were described by the proportion of each category, while continuous variables were characterized using their mean and standard deviation. For PFAS serum concentrations below the LOD, imputation was performed by assigning the value of LOD divided by the square root of 2 [52]. Log‐transformations were applied to serum PFAS concentrations in all inferential analyses to reduce right skewness and better satisfy the regression model assumptions.

Serum PFAS concentrations of the ARFF firefighter study participants were compared to the general U.S. population using data from NHANES—a nationally representative sample of the non‐institutionalized U.S. civilian population using a stratified multistage probability sample that is administered by National Center for Health Statistics, CDC. NHANES subsample weights were applied to produce nationally representative estimates. To determine if there exist significant differences in PFAS concentrations between ARFF firefighters and the general population (NHANES 2017–March 2020), we utilized pooled survey‐weighted multivariable regression models with log‐transformed PFAS concentrations as outcomes. Following prior approaches for pooled analyses, NHANES subsample weights were normalized to sum to the NHANES PFAS sample size, while ARFF firefighters were assigned sampling weights of 1 [53]. We incorporated the original NHANES design variables for primary sampling units and strata, while ARFF firefighters were treated as independent observations with a single common stratum. The model adjusted for covariates, including age, sex, and race/ethnicity [54]. Exposure ratios between ARFF firefighters and NHANES were calculated as adjusted geometric mean ratios by exponentiating the regression coefficients for the study group indicator. Confidence intervals (CIs) for these ratios were derived based on the corresponding coefficient CIs on the log scale. This analysis encompassed PFHxS, n‐PFOS, sm‐PFOS, n‐PFOA, PFNA, PFDA, PFUnDA [55], and the sum of the above PFAS (∑PFAS). Sb‐PFOA and MeFOSAA were not included in inferential analyses due to their relatively low detection frequencies in the ARFF firefighters (2% and 30%, respectively). All survey‐weighted analyses were performed using the *survey* package in R (version 4.5.2).

Multiple linear regression models were used to examine associations between firefighting exposure risk (independent variables) and log‐transformed serum PFAS concentrations (dependent variable) among ARFF firefighters. PFAS that were detected in at least 50% of samples were included in the analysis: PFHxS, n‐PFOS, sm‐PFOS, n‐PFOA, PFNA, PFDA, and PFUnDA, and ∑PFAS. For the regression models of each PFAS, PFAS in drinking water were evaluated only for the corresponding PFAS in serum. For example, detectable PFOS in water was evaluated for detectable concentrations of n‐PFOS and sm‐PFOS in serum. The regression dataset contained 18 samples with missing values. Age was imputed for two samples based on career firefighting years, volunteer firefighting years, and sex. The remaining 16 samples were excluded from the regression analysis.

## 3 Results

### 3.1 Participant Demographics and Exposure Risk Factors

A total of 193 participants from six ARFF fire departments (denoted as departments A‐F) across the continental USA enrolled in this study, with a mean (range) of 32 (4–77) participants per department. Most participants were male (88.5%) and non‐Hispanic white (69.8%), with a mean age of 43 years (Table 1). On average, participants had been working as career firefighters for 16 years and 24% had prior military experience.

**Table 1:** Demographics and exposure factor characteristics (*n* = 193).

	*n* (%)		mean (SD)
Demographics			
Sex		Age (years)	43.1 (10.9)
Male	169 (88.5%)	Career (years)	15.6 (10.8)
Female	22 (11.5%)	Volunteer (years)	2.0 (2.9)
Missing	2		
Race/ethnicity			
Non‐Hispanic White (NHW)	132 (69.8%)		
Hispanic White (HW)	11 (5.8%)		
Black	14 (7.4%)		
Other/multiracial*	32 (16.9%)		
Missing	4		
Military service			
No	146 (76.4%)		
Yes	45 (23.6%)		
Missing	2		
Fire Department			
Department A	29 (15.0%)		
Department B	34 (17.6%)		
Department C	19 (9.8%)		
Department D	77 (39.9%)		
Department E	30 (15.5%)		
Department F	4 (2.1%)		
Firefighting characteristics			
Workplace change in behavior or practice for AFFF use			
No	58 (31.0%)		
Less than 1 year	79 (42.3%)		
More than 1 year	50 (26.7%)		
Missing	6		
Frequency of AFFF use with PPE (number of uses per year)			
0‐ < 5	117 (62.2%)		
5–10	40 (21.3%)		
> 10	31 (16.5%)		
Missing	5		
Use of turnout gear personal protective equipment (PPE) ensemble in a typical year†			
Less	60 (31.6%)		
Moderate	92 (48.4%)		
More	38 (20.0%)		
Missing	3		
Other sources of exposure			
Past employment with AFFF exposure			
No	152 (80.0%)		
Yes	38 (20.0%)		
Missing	3		
Current secondary employment with AFFF exposure			
No	185 (97.4%)		
Yes	5 (2.6%)		
Missing	3		

*Includes two or more races. †Turnout gear use categories were based on the number of occurrences and duration: Less = 1–100 uses with < 50% lasting > 1 h; Moderate = 1–100 uses with ≥ 50% lasting > 1 h or > 100 uses with < 50% lasting > 1 h; More = > 100 uses with ≥ 50% lasting > 1 h.

Twenty percent and 3% of participants reported previous employment with possible AFFF exposure and current secondary employment with possible AFFF exposure, respectively. When performing activities involving AFFF, including transferring AFFF, cleaning equipment contaminated with AFFF, or performing activities to contain AFFF runoff, participants reported using PPE (e.g., gloves) 85%, 84%, and 92% of the time, respectively. Most (62%) participants reported using AFFF, with PPE, less than five times in the past year, while 21% and 16% reported use 5–10 and > 10 times in the past year, respectively.

Use of structural turnout gear in a typical year, evaluated as a combination of frequency of use and time wearing ensembles, resulted in 32% categorized as wearing turnout gear for less time (1‐100 occurrences with < 50% of occurrences for > 1 h), 48% categorized as moderate time in gear (1–100 occurrences with ≥ 50% of occurrences for > 1 h or > 100 occurrences with < 50% of occurrences for > 1 h), and 20% categorized as wearing gear for more time ( > 100 occurrences with ≥ 50% occurrences for > 1 h).

PFAS were detected in tap water at three fire departments (B, C, and F). For Department B, PFBA (3.9 ppt), PFPeA (6.0 ppt), PFHxA (4.8 ppt), PFHpA (2.3 ppt), PFOA (4.8 ppt), PFBS (5.2 ppt), PFHxS (4.2 ppt), and PFOS (18.6 ppt) were detected in the drinking water. For Department F, only PFBS (1.2 ppt) was detectable. For Department C, the filtered tap drinking water did not contain any detectable PFAS, but the unfiltered kitchen sink tap water contained PFBA (1.3 ppt), PFPeA (1.3 ppt), and PFHxA (1.5 ppt). None of the field blank samples contained detectable levels of any measured PFAS.

### 3.2 Comparison of Serum PFAS Concentrations by Fire Department

**Figure 1:**
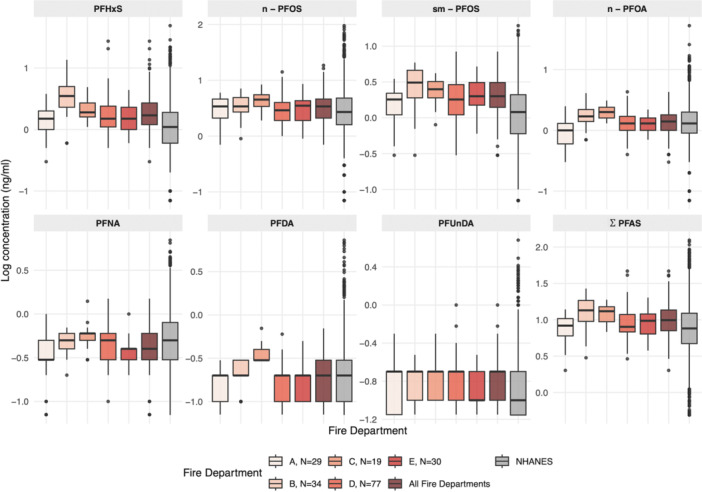
Distribution of log‐transformed serum PFAS concentrations (ng/ml) among ARFF firefighters by fire department (2019–2020) and NHANES participants (2017‐March 2020). Boxplots represent median and interquartile range, not adjusted using age, sex, and race/ethnicity. Fire department F not presented due to small sample size. NHANES distributions are weighted to be nationally representative.

Of the nine PFAS measured in serum, n‐PFOA, n‐PFOS, sm‐PFOS, and PFHxS were detected in 100% of participants, with PFNA, PFDA, and PFUnDA detected in 98%, 94%, and 83%, respectively (Table 2). The geometric mean (GSD) for ∑PFAS analyzed in serum was 9.75 (1.69) ng/mL; the geometric mean concentration by department ranged from 7.59 (1.58) to 24.28 (1.78) ng/mL. The highest concentration was observed in a department with the lowest sample size of only four participants (Department F), with the next highest ∑PFAS concentrations observed at Departments B (12.75 (1.65) ng/mL) and C (12.12 (1.36) ng/mL). The distribution of each PFAS by department is illustrated in Figure 1. Due to the small sample size at Department F, summary statistics are not presented in Table 2 or Figure 1 for this department. Summary statistics for all ARFF study participants are provided in Supplemental Materials (Table S1).

**Table 2:** Serum PFAS concentrations (ng/mL) among ARFF firefighters (2019–2020), ARFF by fire department (A–E*), and the U.S. general population (NHANES 2017–March 2020). Statistics include detection frequency (DF), geometric mean (GM), geometric standard deviation (GSD)**, and adjusted geometric mean ratios comparing ARFF firefighters with NHANES participants***.

	DF NHANES (%)	DF ARFF (%)	GM NHANES (GSD)	GM ARFF (GSD)	GM Dept A (GSD)	GM Dept B (GSD)	GM Dept C (GSD)	GM Dept D (GSD)	GM Dept E (GSD)	Adjusted GM Ratio ARFF vs NHANES $e^{\beta }$ (95% CI)
			*n* = 2676	*n* = 193	*n* = 29	*n* = 34	*n* = 19	*n* = 77	*n* = 30	ARFF *n* = 189, NHANES *n* = 2686
PFHxS	98.5	100.0	1.09 (2.38)	1.89 (2.05)	1.41 (1.80)	3.46 (2.05)	2.06 (1.48)	1.63 (2.01)	1.51 (1.69)	**1.40 (1.24, 1.58)**
n‐PFOS	99.5	100.0	2.87 (2.35)	3.24 (1.81)	2.93 (1.69)	3.40 (1.63)	4.34 (1.50)	2.92 (1.84)	3.14 (1.83)	0.98 (0.89, 1.08)
sm‐PFOS	98.5	100.0	1.21 (2.39)	1.96 (1.94)	1.46 (1.84)	2.77 (1.93)	2.37 (1.50)	1.69 (1.95)	2.02 (1.80)	**1.38 (1.25, 1.53)**
n‐PFOA	99.5	100.0	1.32 (1.99)	1.35 (1.61)	0.87 (1.67)	1.77 (1.52)	1.97 (1.29)	1.30 (1.55)	1.29 (1.37)	0.96 (0.89, 1.05)
sb‐PFOA	6.1	2.1	—	—	—	—	—	—	—	—
PFNA	94.8	98.4	0.46 (2.25)	0.43 (1.68)	0.29 (1.98)	0.46 (1.37)	0.54 (1.44)	0.49 (1.66)	0.35 (1.52)	0.90 (0.80, 1.01)
PFDA	82.2	93.8	0.18 (2.07)	0.19 (1.69)	0.15 (1.65)	0.20 (1.55)	0.37 (1.29)	0.16 (1.69)	0.17 (1.48)	1.03 (0.94, 1.14)
PFUnDA	61.5	83.4	0.12 (1.93)	0.15 (1.70)	0.15 (1.80)	0.14 (1.65)	0.19 (1.63)	0.16 (1.73)	0.12 (1.50)	**1.20 (1.09, 1.33)**
MeFOSAA	50.4	30.1	—	—	—	—	—	—	—	—
∑PFAS	99.9	100.0	7.92 (2.04)	9.75 (1.69)	7.59 (1.58)	12.75 (1.65)	12.12 (1.36)	8.89 (1.68)	8.96 (1.56)	**1.09 (1.01, 1.18)**

*Note:* Bold values indicate a signficant increase relative to NHANES.

*Fire department F not presented due to small sample size

**Included imputed values for < LOD. LOD was 0.1 ng/mL for all PFAS examined. The NHANES sample sizes are presented as unweighted counts, but the GM and GSD were calculated using survey weighted to generate nationally representative estimates.

***Adjusted geometric mean ratios derived from survey‐weighted regression models adjusted for age, sex, and ethnicity. ARFF *n* = 189 in adjusted models due to missing demographic data for four samples.

### 3.3 Comparison of Serum PFAS Concentrations between ARFF and NHANES

After adjusting for age, sex, and ethnicity, serum concentrations were higher (*p* ≤ 0.05) in ARFF firefighters compared with the general population (age 18 and above) represented in NHANES for PFHxS (1.40 fold, 95% CI 1.24, 1.58), sm‐PFOS (1.38, 95% CI 1.25, 1.53), PFUnDA (1.20, 95% CI 1.09, 1.33), and ∑PFAS (1.09, 95% CI 1.01, 1.18) (Table 2). No significant differences were observed for the other PFAS examined.

### 3.4 Associations between Serum PFAS Concentrations in ARFF Firefighters and Exposure Risk Factors

Table 3 summarizes the associations between log‐transformed PFAS concentrations and factors pertaining to participant demographics, firefighting occupation, and select exposure sources using the adjusted exponentiated beta coefficients. This analysis was replicated using the beta coefficients and provided in Supplemental Materials (Table S2). Demographic characteristics included significantly lower serum PFHxS, n‐PFOS, sm‐PFOS, PFDA, and ∑PFAS in females compared with males and lower n‐PFOA in Black participants compared with non‐Hispanic White participants. However, individuals identifying as another race and ethnicity had higher serum PFNA than non‐Hispanic White participants. Age was positively associated with serum sm‐PFOS concentrations, while associations with n‐PFOS and ∑PFAS were directionally positive but did not reach statistical significance.

**Table 3:** Associations between PFAS serum concentrations, demographics, and exposure risk factors (*N* = 177). Values represent adjusted exponentiated beta coefficients ($e^{\beta }$) from multivariable linear regression models, with 95% confidence intervals.

	PFHxS	n‐PFOS	sm‐PFOS	n‐PFOA	PFNA	PFDA	PFUnDA	∑PFAS
(Intercept)	1.11(0.60,2.04)	1.47(0.88,2.44)	0.64(0.37,1.10)	0.91(0.58,1.43)	0.23(0.14,0.38)	0.11(0.07,0.19)	0.11(0.07,0.19)	4.54(2.93,7.03)
Age	1.01(0.99,1.03)	1.02(1.00,1.03)	1.03(1.01,1.04)	1.01(0.99,1.02)	1.01(1.00,1.03)	1.02(1.00,1.03)	1.01(0.99,1.02)	1.02(1.00,1.03)
Female Sex (ref = Male)	0.51(0.38,0.69)	0.50(0.39,0.65)	0.48(0.37,0.62)	0.84(0.68,1.05)	0.80(0.63,1.02)	0.77(0.6,0.99)	0.99(0.76,1.28)	0.59(0.48,0.73)
Race (ref = NHW)								
HW	1.12(0.75,1.65)	0.97(0.7,1.34)	0.85(0.60,1.21)	0.85(0.64,1.14)	0.76(0.55,1.05)	1.16(0.83,1.62)	0.93(0.66,1.31)	0.89(0.67,1.19)
Black	1.07(0.72,1.61)	1.22(0.87,1.71)	1.15(0.80,1.65)	0.71(0.52,0.96)	0.98(0.7,1.37)	0.90(0.63,1.27)	1.37(0.96,1.95)	1.06(0.79,1.42)
Other*	0.93(0.73,1.19)	0.86(0.7,1.06)	0.88(0.70,1.09)	1.08(0.90,1.30)	1.23(1.01,1.51)	1.09(0.89,1.35)	1.17(0.95,1.45)	0.94(0.78,1.12)
Career Firefighter (Years)	1.00 (1.001.02)	1.00(0.98,1.02)	1.00(0.99,1.01)	1.00(0.99,1.02)	1.00(0.99,1.02)	1.00(0.99,1.02)	1.00(0.98,1.01)	1.00(0.98,1.01)
Volunteer Firefighter (Years)	0.98 (0.94, 1.01)	0.98(0.94,1.01)	1.01(0.98,1.05)	1.02(0.99,1.05)	1.01(0.98,1.04)	1.02(0.99,1.05)	1.01(0.98,1.04)	1.01(0.98,1.04)
Any Military Service (ref = No)	1.10 (0.87, 1.40)	1.10(0.87,1.4)	1.19(0.98,1.46)	1.04(0.84,1.29)	0.91(0.76,1.08)	0.93(0.77,1.13)	0.89(0.73,1.09)	1.00(0.82,1.23)
Other occupational AFFF exposure								
Past employment (ref = N)	1.53(1.19,1.96)	1.34(1.09,1.66)	1.27(1.01,1.58)	1.13(0.94,1.36)	0.92(0.75,1.13)	1.04(0.84,1.28)	0.75(0.60,0.93)	1.27(1.06,1.52)
Current secondary employment (ref = No)	0.87(0.46,1.65)	0.68(0.40,1.15)	0.67(0.38,1.18)	0.81(0.50,1.30)	0.61(0.36,1.03)	0.70(0.41,1.21)	0.67(0.38,1.17)	0.70(0.44,1.10)
Water PFAS (ref = ND)	1.79(1.33,2.41)	0.99(0.77,1.27)	1.41(1.08,1.83)	1.42(1.14,1.77)	N/A	N/A	N/A	1.45(1.18,1.78)
Workplace behavior change								
Less than 1 yr (ref = No)	0.77(0.61,0.97)	0.86(0.71,1.05)	0.89(0.72,1.09)	0.85(0.72,1.01)	0.83(0.69,1.00)	0.91(0.75,1.11)	0.95(0.78,1.16)	0.84(0.71,0.99)
More than 1 yr (ref = No)	0.84(0.64,1.10)	0.84(0.67,1.06)	0.83(0.65,1.05)	0.84(0.68,1.03)	0.88(0.71,1.10)	0.78(0.62,0.98)	0.98(0.78,1.24)	0.85(0.70,1.03)
Frequency of AFFF use per year								
5‐10 (ref = <[5])	1.05(0.83,1.32)	1.02(0.84,1.24)	1.05(0.85,1.29)	0.94(0.79,1.11)	1.05(0.87,1.27)	0.97(0.79,1.18)	0.93(0.76,1.14)	1.00(0.85,1.19)
> 10 (ref = <[5])	1.19(0.91,1.56)	1.19(0.95,1.49)	1.15(0.9,1.46)	1.13(0.93,1.38)	1.15(0.92,1.43)	1.04(0.83,1.31)	1.06(0.84,1.34)	1.15(0.94,1.39)
Turnout ensemble†								
Moderate (ref = Less)	1.09(0.87,1.36)	1.01(0.84,1.21)	1.06(0.87,1.29)	1.10(0.93,1.30)	1.12(0.94,1.34)	1.01(0.84,1.21)	1.12(0.92,1.35)	1.10(0.94,1.30)
More (ref = Less)	1.09(0.82,1.45)	1.05(0.83,1.33)	1.12(0.87,1.44)	1.11(0.90,1.37)	1.26(1.00,1.58)	1.06(0.84,1.34)	1.13(0.88,1.43)	1.14(0.93,1.40)

*Note:* Dark blue shading represents *p* ≤ 0.05; light blue shading represents *p* ≤ 0.10 or ≤ 0.05 where 95% CI crosses 1.00.

non‐Hispanic White (NHW), Hispanic White (HW), aqueous film forming foam (AFFF) *Includes two or more races. †Turnout gear use categories were based on the number of occurrences and duration: Less = 1–100 uses with < 50% lasting > 1 h; Moderate = 1–100 uses with ≥ 50% lasting > 1 h or > 100 uses with < 50% lasting > 1 h; More = > 100 uses with ≥ 50% lasting > 1 h.

Frequency of AFFF use in a typical year was not significantly associated with serum concentration of any PFAS (Table 3). However, significant associations were observed between some serum PFAS and characteristics of the participant's current occupation as an ARFF firefighter. Compared with no workplace change in behavior or practice regarding AFFF use, serum PFDA was statistically lower (*p* ≤ 0.05) in participants reporting a change in behavior, policy, or practice that occurred more than 1 year ago. Participants reporting a change within the last year had statistically lower serum PFHxS and ∑PFAS concentrations (*p* ≤ 0.05), while PFNA concentrations were lower, although the 95% confidence interval included the null. Participants primarily reported changes in behavior and policies related to the use of PPE, general precautions when using AFFF, and increased education regarding risks from exposure to AFFF. Conversely, serum PFNA in firefighters who were categorized as wearing turnout gear more frequently was slightly higher (*p* ≤ 0.05) compared with firefighters who were categorized as wearing turnout gear less, although the lower 95% CI overlapped with the null.

Factors related to other sources of exposure included detection of PFAS in fire department tap water and previous employment with known exposure to AFFF. Detection of PFAS in tap water was associated with higher serum concentrations of PFHxS, sm‐PFOS, n‐PFOA, and ∑PFAS. While information on industry and occupation was unavailable, past employment in an occupation involving AFFF exposure was positively associated with serum PFHxS, n‐PFOS, sm‐PFOS, and ∑PFAS, but inversely associated with PFUnDA (Table 3).

## 4 Discussion

Participants from ARFF stations included in this study had elevated serum concentrations of PFHxS, sm‐PFOS, PFUnDA, and ∑PFAS compared to background concentrations in the U.S. population. These elevated concentrations were driven by firefighters from three departments where some PFAS were also detected in drinking or tap water, although water concentrations were below the interim EPA health advisory levels at the time of data collection [56].

Consistent with the literature, in our analysis, serum concentrations of n‐PFOS, sm‐PFOS, and PFHxS were statistically higher in ARFF firefighters reporting previous employment with occupational AFFF exposure. Elevated serum PFOS and PFHxS have been observed in other firefighting populations with known AFFF exposure. Australian firefighters presented a significant exposure‐response association between years of occupational exposure to AFFF and serum PFOS and PFHxS concentration [20]. In that population of Australian firefighters, participants who began their career during or after the phase out of older AFFF formulations had PFOS serum concentrations similar to or slightly above the general population. Another more recent study of Australian firefighters found that individuals who began working after AFFF was replaced had lower PFOA, PFHxS, and PFOS concentrations than those who began working prior to its replacement [22]. In Finland, serum PFAS concentrations increased after AFFF training sessions, with the greatest increases observed for PFHxS and PFOS [25]. However, while our current study focused on a firefighting subgroup where AFFF was maintained and available for use due to Federal Aviation Administration (FAA) regulations, no associations were observed between categorical frequency of AFFF use and serum PFAS concentrations among ARFF firefighters in the factor analysis. This finding may be attributable to the minimally elevated concentrations in this population compared with NHANES, coupled with participants reporting use of AFFF relatively infrequently, with high utilization of PPE, such as gloves, during instances of contact with AFFF.

The effect of workplace behavior or practice on exposure reduction can be profound. PFHxS, PFNA, PFDA, and ∑PFAS concentrations were lower in participants who reported a change in workplace behavior, policy, or practice, suggesting workplace practices have the potential to substantially affect worker exposure to PFAS. Integration of administrative controls (e.g. requiring training prior to completing a process) is effective for reducing contact with a chemical hazard and establishing work practices that reduce the potential for exposure [57, 58]. PPE, when used consistently and correctly, can reduce exposure risk; however, it highly depends on proper utilization and workplace safety culture. While not reported as a leading change in behavior or policy, engineering controls (e.g. enclosing AFFF transfer procedures) can also be highly effective at reducing exposure and may be considered among the options for control mechanisms. Studies based in fire service populations have demonstrated a positive association between safety‐specific leadership in the fire service and safety motivation, including the use of PPE [59].

PFAS contamination of ground and drinking water sources has been well documented following the use of AFFF, particularly for long‐alkyl‐chain PFAS [60]. The FAA requires airports certified under 14 CFR 139.317 “Part 139” to only use foams listed by NAVSEA on the Qualified Products List (QPL), also referred to as “MILSPEC” foams. Until 2024, the MILSPEC QPL did not contain approved fluorine‐free Class B foams and the transition from AFFF to F3 products is anticipated to take time, particularly given the potential financial and logistical complexities involved with cleaning or replacement of foam equipment and replacing large stores of AFFF, such as those maintained at DoD installations, U.S. airports, and most fuel and oil refineries. In our previous analyses, Arizona firefighters [18, 19] exhibited higher serum concentrations of PFOS and PFHxS. These same PFAS are also elevated in populations exposed to groundwater contamination from AFFFs [61].

More frequent turnout gear use was suggestive of an association with higher PFNA within the ARFF cohort, suggesting that occupational sources may contribute to PFNA exposure. Since time wearing turnout gear may function as a surrogate for time spent at live‐fire response, occupational sources may additionally include PFAS present in the fire environment, either directly or through deposition of PFAS combustion byproducts on the gear. The U.S. National Institute for Standards Technology detected PFNA in 7 of 20 turnout gear textiles analyzed for PFAS [9] as well as carboxylate precursors, such as fluorotelomer alcohols, with the potential to transform into PFNA [8, 9]. Some other recent studies have detected PFAS in turnout gear samples [11, 32]. While studies of dermal absorption of PFAS are limited, research characterizing absorption of other long‐alkyl‐chain carboxylates has demonstrated dermal absorption potential [12, 62, 63]. Additionally, exposure may occur through inhalation or ingestion routes from PFAS released from turnout gear into the work environment. Young et al. reported that dust from gear locker areas of 15 fire stations in Massachusetts contained higher absolute median concentration and proportion of seven PFAS, including PFNA, compared with other areas of the station [13].

Our study draws on numerous strengths. The relatively large sample size comprising multiple airports across different U.S. geographic regions is a major strength and adds to the robust interpretation of the data. However, the relatively small sample sizes for certain departments, particularly Department F, limit the generalizability of the findings. The combination of the FFCCS enrollment survey and PFAS‐specific survey enabled exposure risk factor analyses not previously reported in the literature. Serum was analyzed for PFAS at CDC with methods that are directly comparable to those used for the analyses of NHANES samples. While the CDC method for analyzing serum is generally limited to long‐alkyl‐chain PFAS with relatively long half‐lives and do not include shorter‐alkyl‐chain or precursor PFAS that are present in newer AFFF formulations or EPA methods used for water analysis, which include 29 PFAS, shorter‐alkyl‐chain PFAS were detected in relatively few water samples. The collection of fire station drinking water at the time of blood sample collection is another notable strength of the study. Because drinking water is considered the primary source of exposure for the general public, these data allowed characterization of potential background exposure at each of the six fire departments. However, there are also several notable limitations. Water samples may not be representative of PFAS in residential drinking water for participants where their residential source differed from that of the fire station. The exposure factor analysis relied on survey data which are subject to reporting and recall bias. Due to considerations for participant time and concern regarding survey fatigue, the PFAS‐specific survey was limited in length and did not include a robust assessment of non‐occupational sources of PFAS or factors that may affect elimination, such as blood or plasma donation [64]. Additionally, compared to the historically prominent long‐alkyl‐chain PFAS, newer‐generation fluorinated PFAS generally consist of shorter‐alkyl‐chain (e.g. sulfonic acids with carbon chain length less than six or carboxylic acids with carbon chain length of six or fewer, aka “C6”) or precursor (e.g. fluorotelomer alcohol) chemical formulations. These chemistries (or their degradants) are still highly persistent in the environment; however, they often exhibit a shorter half‐life in the body and are generally considered less bioaccumulative than their long‐alkyl‐chain counterparts [65]. Characterization of the presence of these other PFAS in firefighter populations is limited and was not evaluated in this study. It's also important to note that some precursor formulations can metabolize into PFCAs in the body, so accumulation is an ongoing concern. Lastly, this study was not able to evaluate differences related to specific workplace behavior changes, such as routine laundering of turnout gear, the use of preliminary exposure reduction, or wearing gloves during AFFF use. Future research may benefit from building on the exposure factors assessed in this study and expanding the information collected in the survey responses, including the evaluation of individual workplace behavior changes.

## 5 Conclusions

In this study of ARFF firefighters from six U.S. airport fire departments, we observed elevated serum concentrations of PFHxS, sm‐PFOS, and PFUnDA compared to the general population. While some PFAS were detected in tap water from three of the six fire departments, specific PFAS detected in both water and firefighter serum only overlapped in one department. Past employment in an occupation with AFFF exposure was positively associated with serum concentrations of n‐PFOS, sm‐PFOS, and PFHxS; however, despite the presence and use of AFFF at all fire departments, no association was observed between serum PFAS and categorical frequency of current AFFF use. Among other exposure factors assessed in this study, changes in workplace behaviors or practices regarding AFFF use were associated with lower serum concentrations of PFHxS and ∑PFAS, with directionally similar but non‐significant reductions observed for PFNA and PFDA. Future research is important to better understand the contribution of non‐AFFF exposure pathways as well as specific exposure reduction strategies employed by fire departments.

## Author Contributions

Conceptualization: Miriam M. Calkins and Jefferey L. Burgess. Methodology: Miriam M. Calkins, Antonia M. Calafat, Gavin P. Horn, Judith M. Graber, Alberto J. Caban‐Martinez, Julianne Cook Botelho, Alexander C. Mayer, Yiwen Liu, L.W., Jefferey L. Burgess. Formal analysis: Yiwen Liu. Investigation: All authors. Writing – original draft preparation: Miriam M. Calkins. Writing – review and editing: all authors. Visualization: Yiwen Liu. Supervision, Miriam M. Calkins, Antonia M. Calafat. Project administration: Miriam M. Calkins, Shawn C. Beitel. Funding acquisition: Miriam M. Calkins, Jefferey L. Burgess. All authors have read and agreed to the published version of the manuscript.

## Disclosure

The findings and conclusions in this paper are those of the authors and do not necessarily represent the official position of the National Institute for Occupational Safety and Health (NIOSH) or the Centers for Disease Control and Prevention (CDC). Mention of trade names and commercial products does not constitute endorsement or recommendation for use by NIOSH or CDC.

## Ethics Statement

This study was reviewed and approved by the University of Miami Institutional Review Board (20170997) (See 45 C.F.R. part 46.114; 21 C.F.R. part 56.114), and participating institutions established reliance agreements. Written informed consent was obtained from all subjects involved in the study.

## Conflicts of Interest

The authors declare no conflicts of interest.

## Supporting information

Supporting File:

## Data Availability

Data are available upon reasonable request. Data requests will be reviewed by the study's Oversight and Planning Board to address firefighter concerns prior to determination of sharing de‐identified data. Contact the corresponding author with requests.The data that support the findings of this study are available on request from the corresponding author. The data are not publicly available due to privacy or ethical restrictions.
