# Supplementary material for: Serum PFAS in Aircraft Rescue and Firefighting (ARFF) Firefighters From Six U.S. Airport Fire Departments
Source: Am J Ind Med. 2026 Apr 26;69(7):512–24. doi: 10.1002/ajim.70084 (PMC13231396; doi:10.1002/ajim.70084)
Supplement: Supplementary file 1 — Supporting File: [file AJIM-69-512-s001.docx]

**SUPPLEMENTAL MATERIALS**

Serum PFAS in Aircraft Rescue and Firefighting (ARFF) Firefighters from Six U.S. Airport Fire Departments

| **Table S1**. Serum PFAS concentrations (ng/ml) for full ARFF study participants (2019–2020; N=193). Statistics include detection frequency (%), geometric mean (GM), geometric standard deviation (GSD), and summary statistics. | | | | | | | |
| --- | --- | --- | --- | --- | --- | --- | --- |
| PFAS | % | GM | GSD | 50th | 95th | min | max |
| PFHxS | 100% | 1.89 | 2.05 | 1.70 | 7.04 | 0.30 | 27.3 |
| n-PFOS | 100% | 3.24 | 1.81 | 3.40 | 8.48 | 0.70 | 18.5 |
| sm-PFOS | 100% | 1.96 | 1.94 | 2.00 | 5.20 | 0.30 | 8.40 |
| n-PFOA | 100% | 1.35 | 1.61 | 1.40 | 2.88 | 0.30 | 4.30 |
| sb-PFOA | 2% | -- | -- | -- | -- | -- | -- |
| PFNA | 98% | 0.43 | 1.68 | 0.40 | 0.94 | <LOD | 1.50 |
| PFDA | 94% | 0.19 | 1.69 | 0.20 | 0.40 | <LOD | 0.70 |
| PFUnDA | 83% | 0.15 | 1.70 | 0.20 | 0.30 | <LOD | 1.00 |
| MeFOSAA | 30% | -- | -- | -- | -- | -- | -- |
| ∑PFAS | 100% | 9.75 | 1.69 | 9.87 | 21.32 | 2.01 | 46.70 |
| LOD was 0.1ng/ml for all PFAS examined.  Included imputed values for <LOD | | | | | | | |

**Table S2**. Associations between PFAS serum concentrations, demographics, and exposure risk factors (N=177). Values represent beta coefficients ($\beta$) from multivariable linear regression models, with 95% confidence intervals.

|  | | **PFHxS** | **n-PFOS** | **sm-PFOS** | **n-PFOA** | **PFNA** | **PFDA** | **PFUnDA** | **∑PFAS** |
| --- | --- | --- | --- | --- | --- | --- | --- | --- | --- |
| (Intercept) | | 0.10 (-0.51, 0.72) | 0.38 (-0.13, 0.89) | -0.45 (-0.99, 0.10) | -0.10 (-0.55, 0.36) | -1.47 (-1.97, -0.97) | -2.19 (-2.71, -1.67) | -2.17 (-2.70, -1.64) | 1.51 (1.07, 1.95) |
| Age | | 0.01 (-0.01, 0.03) | 0.02 (0.00, 0.03) | 0.02 (0.01, 0.04) | 0.01 (-0.01, 0.02) | 0.01 (-0.00, 0.03) | 0.02 (0.00, 0.03) | 0.01 (-0.01, 0.02) | 0.02 (0.00, 0.03) |
| Female Sex (ref = Male) | | -0.67 (-0.97, -0.38) | -0.68 (-0.93, -0.44) | -0.74 (-1.01, -0.48) | -0.17 (-0.39, 0.05) | -0.23 (-0.47, 0.02) | -0.27 (-0.52, -0.02) | -0.01 (-0.27, 0.25) | -0.52 (-0.73, -0.31) |
| Race (ref = NHW) | |  |  |  |  |  |  |  |  |
|  | HW | 0.11 (-0.28, 0.50) | -0.03 (-0.36, 0.30) | -0.16 (-0.51, 0.19) | -0.16 (-0.45, 0.13) | -0.27 (-0.59, 0.05) | 0.15 (-0.19, 0.48) | -0.07 (-0.42, 0.27) | -0.12 (-0.40, 0.17) |
|  | Black | 0.07 (-0.34, 0.48) | 0.20 (-0.14, 0.53) | 0.14 (-0.22, 0.50) | -0.34 (-0.64, -0.04) | -0.02 (-0.35, 0.31) | -0.11 (-0.46, 0.24) | 0.31 (-0.04, 0.67) | 0.06 (-0.23, 0.35) |
|  | Other* | -0.07 (-0.32, 0.18) | -0.15 (-0.36, 0.06) | -0.13 (-0.35, 0.09) | 0.08 (-0.11, 0.26) | 0.21 (0.01, 0.41) | 0.09 (-0.12, 0.30) | 0.16 (-0.05, 0.37) | -0.06 (-0.24, 0.11) |
| Career Firefighter (Years) | | 0.00 (-0.02, 0.02) | -0.00 (-0.01, 0.01) | 0.00 (-0.01, 0.02) | 0.00 (-0.01, 0.02) | 0.00 (-0.01, 0.02) | -0.00 (-0.02, 0.01) | -0.00 (-0.02, 0.01) | 0.00 (-0.01, 0.02) |
| Volunteer Firefighter (Years) | | -0.02 (-0.06, 0.01) | 0.01 (-0.02, 0.04) | 0.02 (-0.01, 0.05) | 0.01 (-0.02, 0.04) | 0.02 (-0.01, 0.05) | 0.01 (-0.02, 0.04) | 0.01 (-0.02, 0.04) | 0.01 (-0.02, 0.04) |
| Any Military Service (ref = No) | | 0.10 (-0.14, 0.34) | 0.18 (-0.02, 0.38) | 0.04 (-0.17, 0.25) | -0.10 (-0.27, 0.08) | -0.07 (-0.26, 0.12) | -0.11 (-0.31, 0.09) | -0.00 (-0.20, 0.20) | 0.09 (-0.08, 0.26) |
| Other occupational AFFF exposure | |  |  |  |  |  |  |  |  |
|  | Past employment (ref = N) | 0.42 (0.17, 0.67) | 0.30 (0.09, 0.50) | 0.24 (0.01, 0.46) | 0.12 (-0.07, 0.31) | -0.09 (-0.29, 0.12) | 0.04 (-0.18, 0.25) | -0.29 (-0.51, -0.07) | 0.24 (0.06, 0.42) |
|  | Current secondary employment (ref = No) | -0.14 (-0.78, 0.50) | -0.39 (-0.92, 0.14) | -0.40 (-0.97, 0.16) | -0.21 (-0.69, 0.26) | -0.49 (-1.02, 0.03) | -0.35 (-0.90, 0.19) | -0.40 (-0.96, 0.16) | -0.36 (-0.82, 0.09) |
| Water PFAS (ref = ND) | | 0.58 (0.28, 0.88) | -0.01 (-0.26, 0.24) | 0.34 (0.08, 0.61) | 0.35 (0.13, 0.57) | N/A | N/A | N/A | 0.37 (0.17, 0.57) |
| Workplace behavior change | |  |  |  |  |  |  |  |  |
|  | Less than 1yr (ref = No) | -0.26 (-0.49, -0.03) | -0.15 (-0.34, 0.04) | -0.12 (-0.32, 0.08) | -0.16 (-0.33, 0.01) | -0.19 (-0.37, -0.00) | -0.09 (-0.28, 0.10) | -0.05 (-0.24, 0.15) | -0.17 (-0.34, -0.01) |
|  | More than 1yr (ref = No) | -0.17 (-0.45, 0.10) | -0.17 (-0.40, 0.06) | -0.19 (-0.43, 0.05) | -0.18 (-0.38, 0.03) | -0.12 (-0.34, 0.10) | -0.25 (-0.48, -0.02) | -0.02 (-0.25, 0.22) | -0.17 (-0.36, 0.03) |
| Frequency of AFFF use per year | |  |  |  |  |  |  |  |  |
|  | 5-10 (ref = <5) | 0.05 (-0.19, 0.28) | 0.02 (-0.17, 0.22) | 0.05 (-0.16, 0.26) | -0.07 (-0.24, 0.11) | 0.05 (-0.14, 0.24) | -0.03 (-0.23, 0.16) | -0.08 (-0.28, 0.13) | 0.00 (-0.16, 0.17) |
|  | >10 (ref = <5) | 0.18 (-0.09, 0.45) | 0.17 (-0.05, 0.40) | 0.14 (-0.10, 0.38) | 0.12 (-0.08, 0.33) | 0.14 (-0.08, 0.36) | 0.04 (-0.19, 0.27) | 0.06 (-0.17, 0.30) | 0.14 (-0.06, 0.33) |
| Turnout ensemble† | |  |  |  |  |  |  |  |  |
|  | Moderate (ref = Less) | 0.09 (-0.14, 0.31) | 0.01 (-0.18, 0.19) | 0.06 (-0.14, 0.25) | 0.09 (-0.07, 0.26) | 0.11 (-0.07, 0.29) | 0.01 (-0.18, 0.19) | 0.11 (-0.08, 0.30) | 0.10 (-0.06, 0.26) |
|  | More (ref = Less) | 0.09 (-0.20, 0.37) | 0.05 (-0.19, 0.29) | 0.11 (-0.14, 0.37) | 0.10 (-0.11, 0.32) | 0.23 (0.00, 0.46) | 0.06 (-0.18, 0.29) | 0.12 (-0.12, 0.36) | 0.13 (-0.07, 0.34) |
